# Measuring concern about smile appearance among adults

**DOI:** 10.1093/ejo/cjae053

**Published:** 2024-10-21

**Authors:** Bianca Nubia Souza Silva, Lucas Arrais Campos, Bianca Gonzalez Martins, João Marôco, Timo Peltomäki, Juliana Alvares Duarte Bonini Campos

**Affiliations:** Department of Morphology and children´s clinics, School of Dentistry of Araraquara, São Paulo State University (UNESP), Rua Humaitá 1168, 14801-385, Araraquara, Brasil; Department of Morphology and children´s clinics, School of Dentistry of Araraquara, São Paulo State University (UNESP), Rua Humaitá 1168, 14801-385, Araraquara, Brasil; Faculty of Medicine and Health Technology, Tampere University, Kalevantie 4, 33100, Tampere, Finland; Department of Ear and Oral Diseases, Tampere University Hospital, Elämänkatu 2, 33520, Tampere, Finland; Faculty of Health Sciences, Institute of Dentistry, University of Eastern Finland, Yliopistonrinne 3, 70211, Kuopio, Finland; Department of Biological Sciences, School of Pharmaceutical Sciences, São Paulo State University (Unesp), Rodovia Araraquara Jaú Km 01 (s/n), 14800-903, Araraquara, São Paulo, Brasil; William James Center for Research (WJCR), ISPA—Instituto Universitário, Rua Jardim do Tabaco 34, 1149-041, Lisbon, Portugal; FLU Pedagogy, Nord University, Universitetsalléen 11, 8026, Bodø, Norway; Faculty of Medicine and Health Technology, Tampere University, Kalevantie 4, 33100, Tampere, Finland; Department of Ear and Oral Diseases, Tampere University Hospital, Elämänkatu 2, 33520, Tampere, Finland; Faculty of Health Sciences, Institute of Dentistry, University of Eastern Finland, Yliopistonrinne 3, 70211, Kuopio, Finland; Department of Oral and Maxillofacial Diseases, Kuopio University Hospital, Puijonlaaksontie 2, 70200, Kuopio, Finland; Department of Biological Sciences, School of Pharmaceutical Sciences, São Paulo State University (Unesp), Rodovia Araraquara Jaú Km 01 (s/n), 14800-903, Araraquara, São Paulo, Brasil

**Keywords:** dental esthetics, psychometry, validation study, structural equation modeling

## Abstract

**Background/Objectives:**

To adapt and estimate the psychometric properties of Utrecht Questionnaire for esthetic outcome assessment in rhinoplasty (OAR) to assess concern about smile appearance and to estimate the influence of demographic characteristics on this concern in adults.

**Material/Methods:**

This was a cross-sectional observational study. Individuals aged between 18 and 40 years participated in the study. The Portuguese version of OAR was adapted for smile assessment in dental practice and was named Questionnaire for Outcome Assessment of Smile Aesthetic (OA-Smile). Data validity was estimated using factorial validity [confirmatory factor analysis (CFA)—CFI, Tucker-Lewis index (TLI), SRMR)] and convergent validity (average variance extracted). Reliability was assessed using the alpha ordinal coefficient (α_ordinal_) and the omega coefficient (ω). A structural model was elaborated to assess the contribution of demographic characteristics to smile appearance concerns. Model fit was evaluated, and the z-test (α = 5%) was used to estimate the significance of the path estimates (β).

**Results:**

2.523 subjects participated in the study [mean age = 32.86 (SD = 11.39) years, 68.1% female]. The factor model of orofacial appearance (OA)-Smile presented an adequate fit to the data [CFA: comparative fit index (CFI) = 0.99, TLI = 0.99, SRMR ≤ 0.05]. Convergent validity (AVE ≥ 0.80) and reliability (α_ordinal_ and ω ≥ 0.85) were adequate. The structural model presented an acceptable fit (CFI = 0.974; TLI = 0.991 and SRMR = 0.053). Women, younger people, single people, people with lower income, people using dental prostheses, undergoing dental treatment, and those who do not like their smile were more concerned about their smile appearance.

**Limitations:**

Nonprobability sampling, online data collection, and cross-sectional design are considered limitations of the study.

**Conclusions/Implications:**

OA-Smile is a suitable scale to assess smile appearance concerns, and the data obtained with this scale were valid and reliable. Demographic characteristics should be considered when measuring concerns about smile appearance.

## Introduction

Physical beauty has a broad conceptual value and can be described by evolutionary, psychosocial, or cultural theories. However, one’s first impression of another person is typically based on physical appearance, including the body and the face [1]. The face is directly related to expressiveness and communication between individuals. It is important to establish individual identity and contribute to social interactions and integration, which may explain why people are concerned about appearance [2, 3]. Orofacial appearance (OA) is one of the components of general body appearance that can impact social and subjective well-being [4–6]. OA may also affect the perception of attractiveness, both by oneself and by others [2, 7]. Therefore, self-perception of OA is psychosocially important, regardless of whether there is a relevant aesthetic or functional clinical impairment [8].

For this reason, the self-perception of OA is one of the four dimensions of oral health-related quality of life [9]. In this context, OA refers to the overall appearance of the face, including the dental, oral, and facial features. Smile appearance is a subset of OA and focuses on the smile components, encompassing the lips, teeth, and gums. Teeth appearance refers solely to the aesthetic aspects of the teeth themselves. The self-perception of OA and its components are subjective aspects and, therefore, may differ, for example, between dentist and patient [10]. Thus, the dentist should attempt to identify the patient’s perception so that the treatment plan can be better aligned with their demands and expectations. This approach can enhance patient satisfaction with the treatment [10], especially in orthodontics, which involves tooth movement to improve the smile appearance. However, objective and clinical assessments by the dentist do not always align with the patient’s expectations. Psychometric scales, known as dental patient-reported outcome measures (dPROMs), are an interesting alternative to measure the patient’s perception [11].

Several dPROMs for measuring OA can be found in the literature. These include Psychosocial Impact of Dental Aesthetics Questionnaire (PIDAQ) [12] and Orofacial Esthetic Scale (OES) [13]. PIDAQ measures the impact of dental aesthetics on an individual’s life and consists of four factors (dental self-confidence, social impact, psychological impact, and aesthetic concerns) [12]. OES was originally proposed as a single-factor scale to measure satisfaction with general OA [13]. Both PIDAQ and OES have proven to be adequate dPROMs for measuring these latent constructs: psychosocial impact and satisfaction, respectively [14–16]. Even so, there is still a need for dPROMs that measure other latent constructs, such as concern. They can provide an exploratory means to gather information about patient demand in treatments focusing on smile appearance, including orthodontics and rehabilitation.

Because the self-perception of OA is a patient-reported outcome (PRO), it is important to use short and simple scales that do not overwhelm respondents and are applicable to clinical practice, research settings, and epidemiological studies [17]. Thus, there is room for the inclusion of new measurement tools that are easy to use and measure concern about smile appearance. Utrecht Questionnaire for esthetic outcome assessment in rhinoplasty (OAR) [18] is a psychometric scale with similar characteristics. This scale measures the concern about the nose appearance as an aesthetic outcome of rhinoplasty [18]. Although it focuses on the nose and is applied to individuals who have undergone rhinoplasty, its adaptation to smile appearance can provide the dentist with information that may contribute to a more individualized and patient-centered treatment plan. Adapting a psychometric scale requires rigorous methodological procedures that demonstrate the validity and reliability of the dPROM for a specific context and sample [19].

Demographic characteristics may influence the perception of OA. Campos *et al*. [15] observed that people with lower economic status, who wear dental prostheses, and dislike their smile have a greater psychosocial impact of dental aesthetics on their lives. These authors [15] also found no significant association between self-perception of OA and sex or age, although other studies have shown that OA had more influence on the lives of women [20, 21] and younger people [22, 23]. Therefore, identifying demographic characteristics that influence concern about one’s smile may also be relevant information for planning more targeted treatments that meet patients’ demands and expectations.

This study aimed to adapt OAR to measure concerns about smile appearance, named Questionnaire for Outcome Assessment of Smile Aesthetic (OA-Smile), and estimate the psychometric properties of OA-Smile when applied to adults. In addition, the influence of demographic characteristics on the concern about smile appearance was investigated.

## Methods

### Study design and sampling

This was a cross-sectional observational study with a nonprobability sampling design conducted in two phases: convenience sampling and snowball sampling. Adults of both sexes between the ages of 18 and 40 years participated in the study. The age was limited to 40 years to minimize the effect of this variable on the results. The perception of OA can differ between young and mature adults, with the literature reporting that young people may experience a greater impact of OA on their lives due to their OA [22, 23]. The sample size was estimated according to the proposal of Hair *et al*. [24], who recommend 5 to 10 participants per parameter to be tested in the factor model of the psychometric scale. The model to be tested had 10 parameters (five items and five errors). Thus, the minimum sample size was 50 to 100 participants. However, this is the first time the psychometric properties of the adapted version of OAR to measure concerns about smile appearance (OA-Smile) were being studied. To test the measurement invariance of the factor model and the differential functioning of the items, the analyses were conducted in several independent subsamples (by sex, marital status, use of dental prostheses, dental treatment, liking the own smile, and economic level). Each subsample was therefore planned to have the minimum estimated size. In addition, since the influence of demographic characteristics on smile concern was to be evaluated, it was decided to recruit the largest possible number of participants for the study [25].

### Procedures and ethical aspects

The study was approved by the Research Ethics Committee and included only individuals who consented to participate in the study and signed the informed consent. Because of the Coronavirus Disease 2019 (COVID-19) pandemic, data was collected online from April to September 2021 using a self-reported survey created with LimeSurvey program (LimeSurvey GmbH, Hamburg, Germany; http://www.limesurvey.org). The invitation to participate in the study was initially sent by email to members of the academic community of a University in Brazil (convenience sample). The invitation email contained the aims of the study and a link to the online survey. The first page of the survey presented the informed consent that the participant needed to agree to proceed. The next page was the demographic questionnaire, which collected information on age, sex, marital status, use of dental prostheses, dental treatment, liking the own smile, and economic level. The economic level was classified as E (family monthly income < R$1255), D (R$1255├ 2005), C (R$2005├ 8641), B (R$8641├ 11 262), and A (≥ R$11 262). The exchange rate was U$1.00 = R$5.74 on 2 August 2024, according to the Central Bank of Brazil. The demographic questionnaire was followed by the psychometric scales presented in random order. Subsequently, a snowball sampling strategy was adopted, and participants were asked to forward the link to the online survey to their personal contacts, specifically those not related to academia/university, via email, social networks, or messages.

### Adaptation of OAR to OA-Smile

OAR was originally developed by Lohuis *et al.* [18] to measure the self-perception of the nose appearance aesthetic after rhinoplasty and its impact on the individual’s life. It consists of five items with a 5-point Likert-type response scale (1: not at all to 5: very much) arranged in a unifactorial model. OAR also includes an item in which the participant rates the nose appearance of the nose on an 11-point response scale (0: very ugly to 10: very beautiful). However, this item is used only to determine how the patient rates their nose appearance and is not included in the factor structure. The Portuguese version of OAR was used in the present study [26]. Permission to use the scale was obtained from the original researchers [26] via email before the beginning of the study. The 11-point numerical rating scale result was used to split the participants into two samples according to whether they perceived their smile as ‘beautiful’ (scores ranging from > 5.5 to 10) or ‘ugly’ (scores ranging from 0 to ≤ 5.5).

The adaptation of OAR to measure concern about smile appearance was carried out by a panel of three expert judges, consisting of dentists with clinical experience in smile aesthetics and research experience using psychometric scales. They independently reviewed the original scientific articles [18, 26], the content of each item, the response scale, and the instructions of OAR. They provided suggestions for adapting the scale to measure smile concerns. All three judges agreed that replacing the word ‘nose’ with ‘smile’ was sufficient for this purpose. The researchers adapted the scale based on the judges’ comments (Table 1). This preliminary version of the adapted scale was named Questionnaire for Outcome Assessment of Smile Aesthetic (OA-Smile).

**Table 1.** Portuguese version of UQ for esthetic OAR and the adapted version for measuring concern about smile (OA-Smile).

	Nasal appearance version—OAR^*^	Smile appearance version—OA-Smile
	Instruction: Carefully read each of the questions below and choose the answer that best suits you. The following is the rating of my satisfaction with the appearance of my nose 0 (very ugly)–10 (very beautiful)	Instruction: Carefully read each of the questions below and choose the answer that best suits you. The following is the rating of my satisfaction with the appearance of my smile 0 (very ugly)–10 (very beautiful)
Item		
1	Are you concerned about the appearance of your nose?	Are you concerned about the appearance of your smile?
2	Does this concern bother you often?	Does this concern bother you often?
3	Does this concern affect your daily life (e.g. your work)?	Does this concern affect your daily life (e.g. your work)?
4	Does this concern affect your relationships with others?	Does this concern affect your relationships with others?
5	Do you feel bad about the appearance of your nose?	Do you feel bad about the appearance of your smile?

^*^Lohuis *et al*. [18].

Response scale for the items: 1: not at all, 2: a little, 3: moderately, 4: a lot or often, 5: very much.

### Psychometric properties of OA-Smile

The evidence of validity and reliability of data collected using OA-Smile was verified following the Standards for Educational and Psychological Testing [27]. This study evaluated the content validity, validity based on internal structure, validity based on relationships with external measures, discriminant validity, and validity based on the response process, as described below.

### Content validity

Content validity verifies the adequacy of the grammatical, semantic, and idiomatic terms of the item content and how well the latent construct (concern with smile appearance) is theoretically reflected in the set of items. First, the same panel of three expert judges who participated in the scale adaptation process evaluated the clarity of the items, their practical relevance, and the theoretical appropriateness of the content of the preliminary version of OA-Smile. They considered the content relevant for measuring concern about smile appearance and its impact on individuals’ lives.

A pretest was then conducted on a sample of the target population to verify the incomprehensibility index (II) of the items. II aims to determine whether the participants adequately understood the meaning of the instructions and the content (words and phrases) of the items. II below 20% indicated that the scale was appropriate for participants’ comprehension.

### Validity based on internal structure

Validity based on internal structure verifies how well the relationships among the scale items reflect the latent construct of interest [19, 27]. This analysis aims to ensure that the scale accurately measures what it is intended to measure. First, the psychometric sensitivity of the items was assessed using summary measures [mean, median, and standard deviation (SD)] and the distribution of data (skewness and kurtosis). The absolute values of skewness < 3 and kurtosis < 10 indicated the absence of a severe violation of the normal distribution [19].

Then, factorial validity was assessed using confirmatory factor analysis with the robust Weighted Least Squares Mean and Variance Adjusted (WLSMV). The choice of estimation method was based on the number of points on the response scale (1–5). The fit of the model to the data was assessed using the comparative fit index (CFI), Tucker-Lewis index (TLI), and the standardized root mean residual square (SRMR) [19, 28]. The local fit was also assessed by the factor loadings of the items (λ). Model fit was considered appropriate when CFI and TLI > 0.90, SRMR < 0.08, and λ ≥ 0.50 [19, 28]. The convergent construct validity was estimated using the average variance extracted (AVE), which was considered adequate if AVE ≥ 0.50 [29]. The reliability, which refers to the consistency of the measure by the scale, was estimated using the ordinal coefficient alpha (α) and omega (ω). Values of α and ω ≥ 0.70 were considered adequate [29].

The measurement invariance of OA-Smile was tested to verify if the factor model solution remains consistent across independent samples (random division of the total sample into a test sample and a validation sample) and within subsamples based on demographic variables (sex, marital status, use of dental prostheses, being in dental treatment, liking the own smile, and economic level). Multigroup analysis with CFI difference (∆ CFI) was conducted between two increasingly constrained models. The CFI values of the configural (M0), thresholds (M1), factor loadings (M2), and residuals (M3) models were considered. Invariance was confirmed when the CFI reduction (∆ CFI) between models (M1–M0, M2–M1, and M3–M2) was less than 0.01 [30]. Analyses were performed in the R software [31] using the ‘lavaan’ [32] and ‘semTools’ [33] packages.

### Validity based on the response process

The validity based on the response process was evaluated using Item Response Theory. This analysis examines the probability of a participant endorsing an item based on their level of the latent trait and the difficulty of the item. The information-weighted mean square value (infit: individuals with a latent trait level equal to the item difficulty do not respond as expected) and the unweighted mean square value (outfit: individuals with a latent trait level different from the item difficulty do not respond as expected) were estimated for each subsample given above (in Validity based on internal structure—measurement invariance) using the ‘eRm’ package [34] in the R software [31] and considering the partial credit model (PCM). INFIT and OUTFIT values between 0.5 and 1.5 indicate a reasonable fit of the item to the PCM and were considered productive for the measurement.

Differential item functioning (DIF) was conducted to verify if the items of the scale function similarly across those subsamples. In other words, DIF assesses whether individuals with the same level of the latent trait, but from different subsamples, respond differently to the OA-Smile items. DIF was estimated using ordinal logistic regression based on the likelihood ratio chi-square statistic, with a significance level of 1%. Items with ‘total DIF effect’ (*P* < .01) were considered nonequivalent. McFadden’s and Nagelkerke’s pseudoR^2^ were used [35] and effect sizes < 0.13 were considered negligible [36], i.e. they were not used in the model. DIF and pseudoR^2^ analyses were performed in the ‘lordif’ package [35] of the R software [31].

### Validity based on relationships with external measures

This validity examined how the OA-Smile is related to other established measures of similar or different latent constructs [27]. For that, the Portuguese versions of OES [16] and the OAR [26] were used. OES measures the individual’s satisfaction with their OA. It is an unifactorial scale and consists of seven items with an 11-point response scale (0: very dissatisfied; 10: very satisfied). OA-Smile is expected to have a negative and significant correlation with OES (negative convergent validity), i.e. the lower the satisfaction with the appearance of orofacial aesthetics, the greater the concern about the smile appearance. OAR and OA-Smile were used to estimate discriminant validity, with a low-to-moderate correlation expected between the two variables, as the scales assess independent physical components of the face and are not necessarily strongly correlated.

### Discriminant criterion validity

Discriminant criterion validity was evaluated to estimate whether the scale can identify different groups defined by a given demographic criterion. We defined the groups (subsamples) based on denture use (0 = no; 1 = yes), current dental treatment (0 = no; 1 = yes), and liking the own smile (0 = no, 1 = yes). After checking the measurement invariance of OA-Smile for the different groups, the comparison of smile appearance concern scores between them was performed using analysis of variance (ANOVA). The assumptions of normality and homoscedasticity were tested. The data of all groups presented non-severe violations of normality (skewness < 3, kurtosis < 10) and heteroscedasticity (Levene’s test: *P* < .05). Therefore, Welch’s ANOVA was conducted. A significance level of 5% was adopted. Discriminant criterion validity was established if the difference between group scores was statistically significant.

### Structural model

A structural model was elaborated to estimate the influence of demographic characteristics on concern about the smile appearance. The variables sex (0 = male, 1 = female), marital status (0 = single, 1 = married), economic level (0 = C/D/E, 1 = B/A), use of dental prostheses (0 = no; 1 = yes), being in a dental treatment (0 = no, 1 = yes), and liking the own smile (0 = no, 1 = yes) were the independent variables. The concern about smile appearance (OA-Smile) was the dependent variable of the model. The fit of the structural model was evaluated using the WLSMV estimator, and the goodness of fit was assessed based on the CFI, TLI, and SRMR indices. The hypothetical path estimates (standardized β) were estimated and evaluated using the z-test and a significance level of 5%. This analysis was performed in the R software [31] using the ‘lavaan’ [32] and ‘semTools’ [33] packages.

## Results

### Pretest

Thirty individuals participated in the pretest [56.7% women, mean age=28.2 (SD = 4.6) years]. No item or word/concept was reported as incomprehensible by participants (II = 0%), so there was no need to rephrase the content. Therefore, participants’ understanding of the items was considered adequate, confirming the content validity of OA-Smile for measuring the concern about smile appearance. The preliminary version used in the pretest was considered the final version of OA-Smile (Table 1).

### Psychometric properties of the OA-Smile

A total of 2523 individuals participated in the study (age: mean = 32.86, SD = 11.39 years; 68.1% female), of whom 58.4% were single and 36.8% married. In terms of economic level, 21.3% belong to level A, 16.2% to level B, 44.7% to C, 10% to D, and 7.8% to E. Most participants reported not undergoing dental treatment (80.4%) and liking their smile (77.9%).

**Figure 1.**
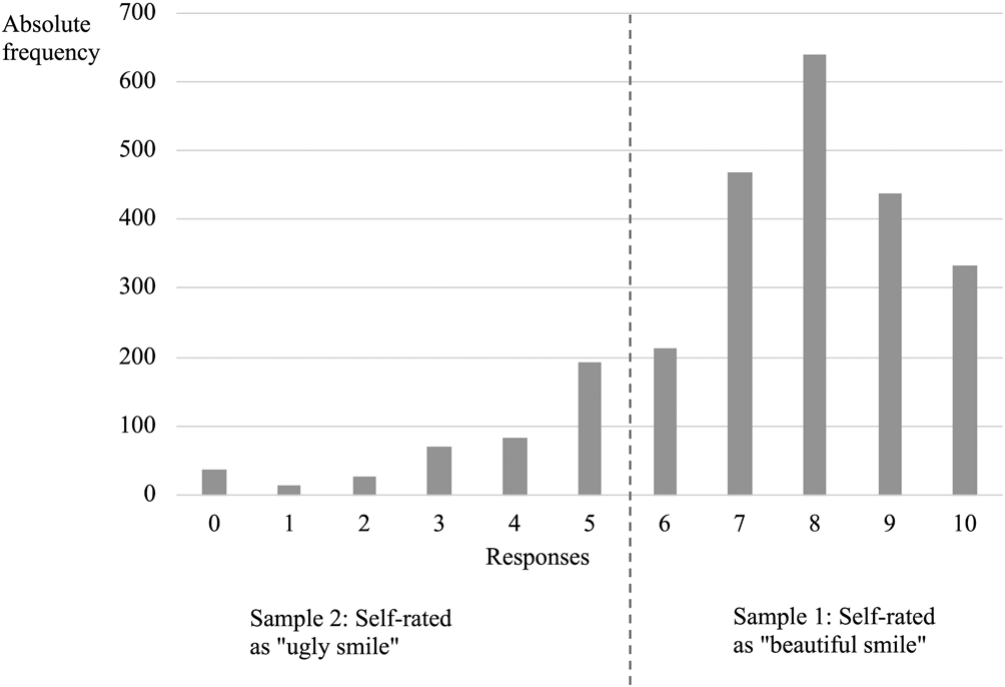
Distribution of participant responses to the 11-point numerical rating scale (0–10) for self-assessment of smile appearance from Questionnaire for OA-Smile.

Figure 1 shows the distribution of participants based on responses to the 11-point numerical rating scale of OA-Smile. Most participants (83.1%, *n* = 2.091) rated their smile as ‘beautiful’ (> 5.5 to 10) and 16.9% (*n* = 425) as ‘ugly’ (scores ranging from 0 to ≤ 5.5).

### Validity based on internal structure

The data indicated that items of OA-Smile presented adequate psychometric sensitivity for each subsample. The model had adequate fit, convergent validity, and reliability for all subsamples (Table 2). Strict invariance was found between models for all characteristics assessed (Table 2).

**Table 2.** Psychometric indicators of OA-Smile for the subsamples.

	CFA^*^				Reliability		ΔCFI		
Subsample	CFI	TLI	SRMR	AVE	ω	ɑ	M1–M0	M2–M1	M3–M2
Test	0.995	0.991	0.029	0.839	0.935	0.959			
Validation	0.995	0.990	0.042	0.852	0.935	0.959	<0.001	<0.001	<0.001
Numerical rating scale									
Ugly	0.997	0.994	0.023	0.852	0.941	0.964			
Beautiful	0.994	0.988	0.038	0.843	0.933	0.958	<0.001	<0.001	<0.001
Sex (*n* = 2.505)									
Male	0.998	0.996	0.042	0.839	0.933	0.906			
Female	0.999	0.997	0.031	0.846	0.935	0.910	<0.001	<0.001	<0.001
Marital status (*n* = 2.397)									
Single	0.998	0.996	0.036	0.829	0.933	0.910			
Married	0.998	0.997	0.042	0.861	0.932	0.900	<0.001	<0.001	<0.001
Use of dental prostheses (*n *= 2.517)									
No	0.998	0.997	0.034	0.839	0.932	0.906			
Yes	0.998	0.997	0.054	0.907	0.964	0.940	<0.001	<0.001	<0.001
Dental treatment (*n* = 2.514)									
No	0.998	0.997	0.034	0.838	0.929	0.902			
Yes	0.998	0.996	0.043	0.846	0.943	0.922	<0.001	<0.001	<0.001
Likes the Smile (*n* = 2.517)									
No	0.995	0.990	0.059	0.789	0.927	0.912			
Yes	0.998	0.997	0.054	0.907	0.964	0.940	-0.001	-0.002	<0.001
Economic level (*n* = 2.514)^#^									
Low (C/D/E)	0.998	0.996	0.037	0.845	0.939	0.918			
High (A/B)	0.998	0.996	0.040	0.799	0.900	0.851	<0.001	<0.001	<0.001

^*^CFA = Confirmatory Factor Analysis: CFI = Comparative Fit Index; TLI = Tucker-Lewis Index; SRMR = Standardized root mean residual square; AVE = Average Variance Extracted; ω= omega coefficient; α=ordinal alpha coefficient; ΔCFI = CFI difference; M0 = configural model; M1 = threshold model; M2 = factor loading model; M3 = residuals model. ^#^Economic level: A (monthly family income above R$11 262.00); B (from $8641.00 to 11 261.00); C (from R$2005.00 to 8640.00), D (from R$1255.00 to 2004.00), and E (from 0 to R$1254.00). The exchange rate was U$1.00 = R$5.74 on 2 August 2024, according to the Central Bank of Brazil.

### Validity based on response process

Table 3 shows the item fit statistics of OA-Smile. The fit of OA-Smile items was appropriate for the subsamples according to sex, marital status, use of dental prostheses, dental treatment, liking of own smile, and economic level. Items 2 and 4 presented different responses (DIF) according to sex, but the effect sizes were small (pseudoR² = 0.003 and 0.005, respectively). For marital status, items 1, 4, and 5 had DIF with a low effect size (pseudoR² = 0.004–0.010). DIF was confirmed in items 1 and 5 between groups based on dental treatment and economic level, respectively. The effect sizes were also small (pseudoR² = 0.003 and 0.004). For liking the own smile, most items had DIF, with effect sizes ranging from 0.003 to 0.019. Regarding the use of dental prostheses, the items did not show any DIF.

**Table 3.** Item fit statistics (INFIT and OUTFIT) and DIF analysis results for OA-Smile across subsamples.

Categories—subsamples	Fit, DIF, and pseudo R²	Items				
		OA-Smile 1	OA-Smile 2	OA-Smile 3	OA-Smile 4	OA-Smile 5
Sex—Male/Female	Infit	0.977/1.010	0.583/0.598	0.928/0.810	0.767/0.766	0.756/0.769
	Outfit	0.912/0.947	0.533/0.563	0.995/0.475	0.635/0.724	0.661/0.775
	DIF—*P* χ^2^	.228	<.001	.065	<.001	.123
	R² McFadden	<0.001	0.003	0.002	0.005	<0.001
	R² Nagelkerke	<0.001	0.001	0.001	0.004	<0.001
Marital status—single /married	Infit	0.970/1.046	0.602/0.569	0.891/0.690	0.775/0.795	0.781/0.749
	Outfit	0.909/0.956	0.553/0.543	0.583/0.681	0.795/0.474	0.754/0.738
	DIF—*P* χ^2^	<.001	.065	.002	<.001	<.001
	R² McFadden	0.007	<0.001	0.004	0.010	0.004
	R² Nagelkerke	0.005	<0.001	0.003	0.009	0.003
Dental prostheses—no/yes	Infit	1.001/1.014	0.596/0.586	0.837/0.893	0.782/0.744	0.764/0.703
	Outfit	0.934/0.984	0.555/0.589	0.606/0.721	0.718/0.585	0.754/0.624
	DIF—*P* χ^2^	.196	.263	.783	.813	.322
	R² McFadden	<0.001	<0.001	<0.001	<0.001	<0.001
	R² Nagelkerke	<0.001	<0.001	<0.001	<0.001	<0.001
Dental treatment—no/yes	Infit	0.992/1.047	0.615/0.544	0.813/0.917	0.767/0.804	0.758/0.724
	Outfit	0.926/0.993	0.573/0.512	0.606 /0.645	0.628/1.079	0.747/0.677
	DIF—*P* χ^2^	.759	.820	.505	.492	<.001
	R² McFadden	<0.001	<0.001	<0.001	<0.001	0.003
	R² Nagelkerke	<0.001	<0.001	<0.001	<0.001	0.002
Smile linking—no/yes	Infit	0.857/1.027	0.652/0.599	0.891/0.700	0.907/0.602	0.732/0.836
	Outfit	0.839/0.919	0.633/0.547	0.721/0.518	0.895/0.618	0.720/0.790
	DIF—*P* χ^2^	<.001	.002	<.001	<.001	<.001
	R² McFadden	0.003	0.002	0.006	0.004	0.019
	R² Nagelkerke	0.002	<0.001	0.006	0.004	0.013
Economic level—low /high	Infit	0.998/0.928	0.608/0.572	0.845/0.843	0.793/0.775	0.770/0.771
	Outfit	0.953/0.821	0.571/0.527	0.554/0.775	0.807/0.490	0.737/0.778
	DIF—*P* χ^2^	<.001	.013	.901	.097	.350
	R² McFadden	0.004	0.001	<0.001	0.001	<0.001
	R² Nagelkerke	0.003	<0.001	<0.001	0.001	<0.001

Slash punctuation marks were inserted between the estimates to separate the values obtained for each subsample.

### Validity based on relationships with external measures

Correlation analysis showed adequate negative convergent validity (OA-Smile factor *vs* OES factor: *r* = −0.686; *P* < .001) and discriminant validity (OA-Smile factor vs OAR factor: *r* = 0.371; *P* < .001).

### Discriminant criterion validity

Individuals who use dental prostheses (mean score = 1.98, SD = 1.15, 95% CI = 1.79–2.16), do not like their smile (mean score = 2.61, SD = 1.05, 95% CI = 2.52–2.70), or are undergoing dental treatment (mean score = 2.01, SD = 1.02, 95% CI = 1.91–2.10) showed greater concern about smile appearance compared to those who do not use prostheses (mean score = 1.67, SD = 0.84, 95% CI = 1.64–1.71), like their smile (mean score = 1.43, SD = 0.57, 95% CI = 1.41–1.46), or are not undergoing dental treatment (mean score = 1.61, SD = 0.80, 95% CI = 1.58–1.65). The comparisons between subsamples according to the independent variables revealed significant differences, confirming the discriminant criterion validity of OA-Smile (prostheses use—Welch’s ANOVA: *F* = 10.05, *P* = .002; like their own smile—Welch’s ANOVA: *F* = 641.68, *P* < .001; and dental treatment—Welch’s ANOVA: *F* = 62.47, *P* < .001).

### Structural model

**Figure 2.**
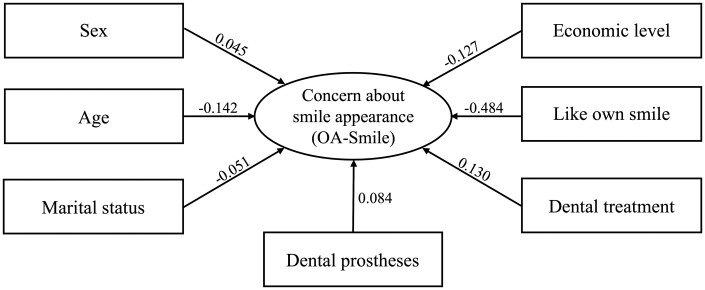
Results of the structural model assessing the influence of demographic characteristics on concern about smile appearance in adults. Notes: The values presented represent the standardized path estimates (β_standardized_). All independent variables presented statistically significant contributions (*P* < .05) to the concern about smile appearance. Sex: 0 = male, 1 = female; age: quantitative measure (in years); marital status: 0 = single, 1 = married; economic level: 0 = C/D/E, 1 = B/A; use of dental prostheses: 0 = no, 1 = yes; being in a dental treatment: 0 = no, 1 = yes; and liking the own smile: 0 = no, 1 = yes. The concern about smile appearance was measured by Questionnaire for OA-Smile.

The structural model with all independent variables showed an acceptable fit (CFI = 0.974; TLI = 0.991, and SRMR = 0.053) with a statistically significant contribution of all independent variables to smile appearance concern (*P* < .05) (Fig. 2). Women, those who were younger, single, had lower income, used dental prostheses, were undergoing dental treatment, and disliked their smile were more concerned about the appearance of their smile.

## Discussion

This study aimed to adapt and assess the psychometric properties of Utrecht Questionnaire (OAR), originally developed to study the aesthetics of rhinoplasty [18], to measure concerns about smile appearance. These processes were conducted in line with international standards [27], and OA-Smile was introduced, serving as an initial tracking tool to better understand these concerns in clinical and research settings. The study was initiated because of the need to consider patients’ perspectives about their smile appearance in dental practice. This is especially important given the increasing demand for aesthetic treatments that affect the smile, such as orthodontics. Although often neglected, adapting a psychometric scale following international recommendations requires rigorous methodological care to ensure that it can measure what it intends to measure when applied to different samples [27].

The validity and reliability of the data obtained by OA-Smile were confirmed when applied to Brazilian adults. This evidence supports recommending this scale for measuring concerns about smile appearance. We hope this study will stimulate further studies using OA-Smile in diverse contexts, e.g. in other cultures and countries. This would facilitate comparisons and the establishment of correlations to promote discussions about OA and, in particular about smile appearance from the patients’ perspective. This is an important topic given the high demand in dental practices focused on aesthetic treatments.

The findings of the present study demonstrated that OA-Smile provides valid and reliable data when applied to different subsamples according to sex, marital status, use of dental prostheses, dental treatment or not, liking the own smile, and economic level. In addition, measurement invariance of OA-Smile factor model was observed across the subsamples. These findings suggest that the scale works similarly in different subsamples to measure the concern about smile appearance. Measurement invariance between groups must be verified whenever sample characteristics change, as comparisons between groups are only possible when invariance is confirmed [37]. Although some items of OA-Smile had DIF between subsamples (IRT analyses), the practical significance was low (pseudoR²). This indicates that the items are generally compatible with the latent trait across each subsample, complementing the results from measurement invariance and suggesting that the scale is suitable for use with subsamples having these different characteristics.

Our second aim was to study the potential influences of demographic characteristics on the concern about smile appearance. Structural Equation Modeling analysis revealed that demographic characteristics have a significant impact on how individuals perceive their smile appearance. The investigation of how self-perception of OA varies between individuals of different sexes and ages is a topic of interest in the literature [15, 38, 39]. Our findings corroborate previous studies [36, 37] which indicate that women and younger individuals perceive the body differently and are more conscious of the appearance of their teeth. In contrast, Campos *et al*. [15] found no significant contribution of age and sex to perceived OA. These authors [15] explained that the scale used in their study measures the psychosocial impact of appearance rather than other latent constructs like satisfaction or concern, which could explain the differing results between studies.

The influence of economic level on OA is consistent with previous studies [15, 16, 40] that found that people with lower economic status place more importance on the appearance of their teeth. This may be related to factors such as oral health status and access to dental products and services [15, 16]. Since these characteristics were not examined in our study, any association between them and the observed results is speculative and should be interpreted with caution. In a related study, Campos *et al*. [5] found that Brazilian adults with lower income experienced a greater psychosocial impact of OA and had less access to aesthetic dental treatment, supporting the latter argument. Our study also found a significant difference in OA based on marital status, which contrasts with the findings of Alhajj *et al*. [41]. These authors [41] reported that single individuals rated their OA more favorably than married individuals, though there were no significant differences in satisfaction with their smiles between the two groups. While Alhajj *et al*. [41] did not find significant differences, they attributed potential variations in self-perception of OA to differing life priorities between married and single people.

In terms of demographic characteristics related to dental practice, participants who underwent dental treatment, used dental prostheses, and reported disliking their own smiles were more concerned about their smile appearance. It suggests that dental treatments, whether prosthetic or otherwise, may significantly impact patients’ psychological well-being [42]. Therefore, professionals should consider the patient’s clinical demands and expectations to achieve treatment success and be aware that professionals’ perceptions may differ from those of the patient [10].

The cross-sectional design is a limitation of the study which does not allow establishing a causal association between the dependent and independent variables. However, this study design is commonly adopted in observational studies on the psychometric properties of scales [43, 44]. Other limitations of the study are the convenience sample and online data collection, in which only participants with internet access were initially recruited at a university in one district state (São Paulo) in Brazil, followed by a snowball strategy. Brazil is a country of continental proportions, with cultural variations across its regions and high social inequality, which makes internet access non-democratic. Thus, the generalization of the results to the entire population may be hindered and should be interpreted with caution. Future national studies using probability sampling, considering different regions of Brazil and socioeconomic levels, are necessary. We clarify that our initial aim was not to obtain results that could be extrapolated to the Brazilian population. Our primary objective was to adapt an existing psychometric scale and assess its psychometric properties to verify its potential applicability in both clinical and research contexts in dentistry. Therefore, the sample and analyses of the study, including measurement invariance analysis across independent samples, were sufficient to address this objective.

In addition, we did not conduct a detailed dental clinical examination of the participants, which could provide even more interesting data. Unfortunately, a clinical examination was not possible because of online data collection, due to the COVID-19 pandemic, a time when social distancing was a public health recommendation that limited clinical care only to emergencies or for a specific clinical indication. Therefore, we suggest that further studies also include clinical examinations to determine associations with smile appearance concerns.

Furthermore, we recognize that certain grammatical structures and wording in OA-Smile may introduce biases in the interpretation and response pattern. However, we decided to retain this language structure to align with the original scale and follow the results of the adaptation steps. This decision was based firstly on the strong evidence of construct validity obtained in the psychometric analyses. Secondly, the study aimed to adapt OAR rather than develop a new scale, which would require a different analytical approach. Lastly, none of the expert judges and study participants provided feedback regarding this issue. Nevertheless, we encourage researchers to consider a thorough investigation of potential interpretability biases, such as social desirability. Future studies conducting both qualitative and quantitative analyses using OA-Smile and other dPROMs are important for identifying relevant constructs and physical components (such as dental characteristics, lips, gum exposure, nose, etc.) related to OA. These studies may help in developing a comprehensive dPROM with minimal response bias. Despite these limitations, the findings presented should promote the use of the OA-Smile in different contexts and samples and contribute to future research and clinical dental practice.

## Conclusion

The OA-Smile is a short, easy-to-use scale that has been shown to produce valid and reliable data when applied to adults and be suitable for use in epidemiologic studies, national surveys, and dental practices to assess specific components of OA, especially in areas such as orthodontics, which works on dental aesthetics. Demographic and clinical characteristics should be considered when assessing smile appearance concerns to tailor treatment to patients’ demands and expectations.

## Data Availability

We have read the journal’s requirements for reporting the data underlying my submission and we inform you that the data sets used and/or analyzed during this study are available from the corresponding author upon request.
